# DEEPCYPs: A deep learning platform for enhanced cytochrome P450 activity prediction

**DOI:** 10.3389/fphar.2023.1099093

**Published:** 2023-04-10

**Authors:** Daiqiao Ai, Hanxuan Cai, Jiajia Wei, Duancheng Zhao, Yihao Chen, Ling Wang

**Affiliations:** Guangdong Provincial Key Laboratory of Fermentation and Enzyme Engineering, Joint International Research Laboratory of Synthetic Biology and Medicine, Ministry of Education, Guangdong Provincial Engineering and Technology Research Center of Biopharmaceuticals, School of Biology and Biological Engineering, South China University of Technology, Guangzhou, China

**Keywords:** cytochrome P450, multi-task FP-GNN, deep learning, online webserver, CYPs inhibitors

## Abstract

Cytochrome P450 (CYP) is a superfamily of heme-containing oxidizing enzymes involved in the metabolism of a wide range of medicines, xenobiotics, and endogenous compounds. Five of the CYPs (1A2, 2C9, 2C19, 2D6, and 3A4) are responsible for metabolizing the vast majority of approved drugs. Adverse drug-drug interactions, many of which are mediated by CYPs, are one of the important causes for the premature termination of drug development and drug withdrawal from the market. In this work, we reported in silicon classification models to predict the inhibitory activity of molecules against these five CYP isoforms using our recently developed FP-GNN deep learning method. The evaluation results showed that, to the best of our knowledge, the multi-task FP-GNN model achieved the best predictive performance with the highest average AUC (0.905), F1 (0.779), BA (0.819), and MCC (0.647) values for the test sets, even compared to advanced machine learning, deep learning, and existing models. Y-scrambling testing confirmed that the results of the multi-task FP-GNN model were not attributed to chance correlation. Furthermore, the interpretability of the multi-task FP-GNN model enables the discovery of critical structural fragments associated with CYPs inhibition. Finally, an online webserver called DEEPCYPs and its local version software were created based on the optimal multi-task FP-GNN model to detect whether compounds bear potential inhibitory activity against CYPs, thereby promoting the prediction of drug-drug interactions in clinical practice and could be used to rule out inappropriate compounds in the early stages of drug discovery and/or identify new CYPs inhibitors.

## 1 Introduction

Cytochrome P450 (CYP) is a superfamily of heme-containing oxidase enzymes found in the smooth endoplasmic reticulum and mitochondria of liver cells and intestines (Neve and Ingelman-Sundberg, 2010). In humans, 57 CYP isoforms have been found to be involved in the oxidative metabolism of various xenobiotics as well as organic endogenous chemicals (Arimoto, 2006; Redlich et al., 2008). Five CYPs isoforms (CYP1A2, CYP2C9, CYP2C19, CYP2D6, and CYP3A4) play crucial roles in approximately 90% of metabolic reactions (Arimoto, 2006). For example, CYP1A2 is responsible for metabolizing about 9% of clinically used drugs, such as antipsychotics and antibiotics (Chen et al., 2017). CYP2C9 contributes to the metabolism of around 15% of all medications, and plays an important role in the metabolism of routinely used pharmaceuticals such as non-steroidal anti-inflammatory drugs (NSAIDs) and warfarin (Daly et al., 2017; Goldwaser et al., 2022, 9). Many therapeutic drugs including clopidogrel, voriconazole, and proton pump inhibitors are metabolized by CYP2C19 (Botton et al., 2021, 19). Furthermore, CYP3A4 and CYP2D6 are responsible for approximately 30% and 20% of clinical drug metabolism, respectively (Bojić et al., 2019). Avoiding the inhibition of drug-metabolizing CYPs is a major challenge in drug development, as inhibiting these CYP isoforms may lead to drug-drug interactions and significant adverse effects. For example, Miguel and Albuquerque illustrated that most antitumor drugs are metabolized by CYP3A4, and their co-administration with antidepressants that inhibit CYP3A4 (e.g., sertraline, fluoxetine, fluvoxamine, and paroxetine) may result in cause loss of efficacy or increased toxicity (Miguel and Albuquerque, 2011). In 2016, over two million significant cases of adverse drug reactions were reported in the United States, of which approximately 26% were judged to be preventable drug-drug interactions (Ho et al., 2016; Le and Le, 2016). For example, Tateishi and coworkers reported the risk of hypoglycemia prompted by the combination of bucolome and glimepiride. Such hypoglycemia may be caused by CYP2C9-mediated drug interactions in combination with bucolome (Tateishi et al., 2021). Therefore, determining the potential for CYPs inhibition can help weed out underperforming drug candidates in the early drug discovery process to reduce the occurrence of termination of drug development programs, drug withdrawal from the market, or restriction of therapeutic use, which is crucial for drug discovery and development.

Various computational approaches have been used to predict or explore CYP-mediated metabolism and inhibition. It is difficult to accurately predict CYP450 inhibitors using structure-based techniques like molecular docking and pharmacophore mapping due to the flexible conformation of CYP450 (Li et al., 2018). In contrast, machine learning (ML)- and deep learning (DL)-based quantitative structure-activity relationship (QSAR) approaches, as the most popular ligand-based methods, are widely utilized to predict CYP450 inhibitors (Tyzack et al., 2016; Xiong et al., 2019). For example, previous studies often used conventional ML (CML) and DL methods to predict different CYP isoform inhibitors with different prediction accuracies (Cheng et al., 2011; Sun et al., 2011). Considering the high sequence homology and structural similarity of binding active sites in the CYP family (Graham and Peterson, 1999; Sun et al., 2011), multi-task models can simultaneously predict inhibitors of different CYP isoforms to provide better predictive power. In 2018, Li et al. (2018) constructed a multi-task DNN model for the five CYP isoenzymes with an average prediction accuracy of 88.7% for the external test datasets. In 2021, Nguyen-Vo et al. (2021). developed iCYP-MFE to further improve the prediction accuracy of CYPs inhibitors using multitask learning and molecular fingerprint-embedded encoding .

**FIGURE 1:**
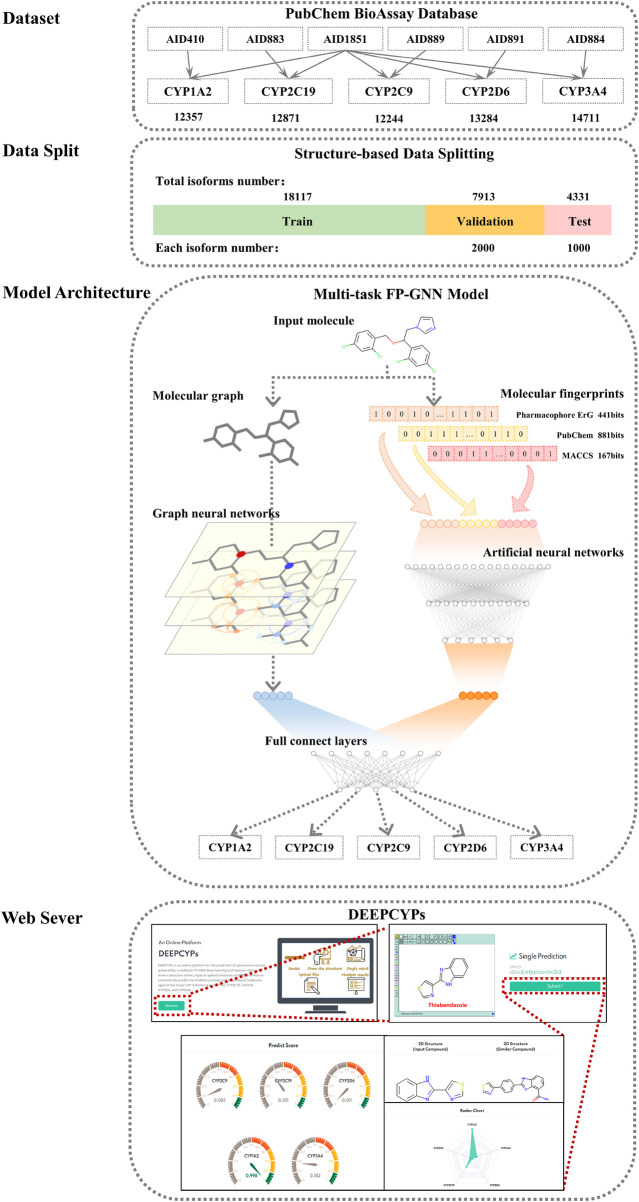
Model construction pipeline.

Recently, we have developed a new DL architecture called FP-GNN (fingerprints and graph neural networks), which combined molecular graph with three molecular fingerprints to improve the ability of deep learning models to predict molecular properties (Cai et al., 2022). Herein, we used a multi-task FP-GNN DL architecture (Figure 1) to construct classification model for predicting the inhibitory activity of molecules against five CYPs (1A2, 2C9, 2C19, 2D6, and 3A4), which achieved state-of-the-art performance compared to baseline predictive models based on four conventional machine learning methods, three deep learning algorithms, as well as two existing models. Moreover, Y-scrambling testing verified that the model results were not by chance. The interpretability analysis provided critical structural fragments associated with CYPs inhibition. An online webserver called DEEPCYPs (https://deepcyps.idruglab.cn/) and its local version python software (https://github.com/idrugLab/FP-GNN_CYP) were established to prioritize compounds in drug discovery to avoid adverse reactions and/or identify new CYP inhibitors.

## 2 Materials and methods

### 2.1 Dataset collection and preparation

We selected the modelling CYP inhibitors datasets reported by (Nguyen-Vo et al., 2021). The modelling datasets contain inhibitors toward five major CYP isoforms (CYP1A2, CYP2C9, CYP2C19, CYP2D6, and CYP3A4). Briefly, chemical data were gathered from six datasets (AID 1851, AID 410, AID 883, AID 889, AID 891, and AID 884) from PubChem BioAssay Database (Wang et al., 2017), which contains 71,456 samples. The dataset AID 1851 contains compounds targeting five isoforms of CYP1A2, CYP2C9, CYP2C19, CYP2D6, and CYP3A4. Samples from datasets AID 410, AID 883, AID 899, AID 891, and AID 884 target compounds of the CYP1A2, CYP2C9, CYP2C19, CYP2D6, and CYP3A4 isoforms, respectively. Such bioassay data come from the same institution (National Center for Advancing Translational Sciences), which ensures consistent experimental protocols for gathering data and minimizing impact of noise. The datasets collected and processed by (Nguyen-Vo et al., 2021) were briefly described as follows: 1) Elimination of inorganics and mixtures; 2) Changing SMILES to canonical SMILES and discarding salts based on XlogP values; 3) Elimination of compounds with multiple structural patterns based on canonical SMILES to avoid incomplete duplication; and 4) Deduplication. Finally, the datasets containing 65,467 samples were obtained. The number of shared compounds was 4,352, which were present in the five data sets. To limit data leakage and make multitask benefits more interpretable, they adopted stringent structure-based data splitting method to generate training, validation, and test sets. (Nguyen-Vo et al., 2021) employed k-mean clustering with k = 6 to divide the samples into six groups. They calculated the within-cluster sum of squared (WSS) errors with different k values by using the Elbow method and chose the k value with the smallest WSS. The validation and test sets for each isoform were created with 2,000 and 1,000 samples, respectively. The 50,467 remaining samples served as training data. The numbers of samples of training data for CYP1A2, CYP2C9, CYP2C19, CYP2D6, and CYP3A4 isoforms were 9,357, 9,244, 9,871, 10,284, and 11,711, respectively. (Nguyen-Vo et al., 2021). The final modelling datasets are freely available at (https://github.com/idrugLab/FP-GNN_CYP).

### 2.2 Multi-task FP-GNN framework and model training protocol

In this study, we used a multi-task FP-GNN framework for predicting inhibition of molecules against the five major CYP isoforms (CYP1A2, CYP2C19, CYP2C9, CYP2D6, and CYP3A4). The technological process of our work is described in Figure 1. Recently, to better forecast molecular properties such as physicochemical properties, biological activities, and ADMET properties, we developed the FP-GNN DL algorithm to concurrently learn molecular graph information and mixed molecular fingerprints information (Cai et al., 2022). For one thing, the fingerprint-based network (FPN) module of the FP-GNN architecture uses an artificial neural network (ANN) to learn information from two substructure-based molecular fingerprints (PubChem FP and MACCS FP) as well as a pharmacophore-based fingerprint (Pharmacophore ErG FP). For another, the graph-based module of the FP-GNN architecture utilizes a spatial graph neural network (GNN) with an attention mechanism to acquire structural information in molecular graphs. The GNN module encodes pre-defined atomic and chemical bond information into molecular graph structure data, and the model communicates the information between surrounding atoms based on the molecular graph structure. It gradually expands over the entire molecular graph. Meanwhile, we use the attention mechanism to update the nodes, focusing on the interactions between surrounding atoms and atoms necessary for the relevant attributes in training. We combine knowledge of all atoms in a molecule to accurately predict its attributes. Finally, FP-GNN architecture employs full connect layers (FCL) to fuse the features from both GNN and FPN paths, and then outputs molecular property prediction results.

The FP-GNN deep learning algorithm (https://github.com/idrugLab/FP-GNN) we developed is a general QSAR modeling method that can be used to build predictive models to predict the properties of molecules, including physicochemical properties, biological activity and ADMET properties. Our lab reported the FP-GNN model achieved the best predictive performance on 13 public datasets (covering biological activities, physicochemical properties, physiology, and toxicity properties), an unbiased LIT-PCBA dataset, and 14 phenotypic screening datasets for breast cell lines (Cai et al., 2022). We successfully selected five compounds using the FP-GNN model to target cycle-dependent family kinase 9 (CDK9) inhibition and demonstrated good anti-cancer activity on eight tumor cells by *in vitro* cell assay. (Zhang et al., 2022). However, most datasets in drug discovery feature significant linkages between subtasks. If only a single-task model is used for modelling, data association information between subtasks would be lost. Therefore, we developed the multi-task FP-GNN framework to prevent data loss from subtasks, which was then successfully used to accurately predict inhibitors of four poly ADP-ribose polymerase (PARP) isoforms (Ai et al., 2022). In this study, we continue to extend the application of the multi-task FP-GNN method in predicting the inhibitory activity of molecules against five CYPs (1A2, 2C9, 2C19, 2D6, and 3A4, Figure 1). Specifically, the multi-task FP-GNN uses a parameter-sharing multi-task learning approach, inherits the molecular graph and molecular fingerprints modules of the single-task FP-GNN model, and finally expands the fusion module into a multi-task output module (Figure 1, middle). All subtasks share the weights of molecular graph and molecular fingerprint modules and extract common features of samples in subtasks. The multi-task output module of FP-GNN accepts the feature information from both GNN (molecular graph path) and ANN (fingerprints path), and then uses the data of different subtasks to optimize the weight of the network, and finally outputs the specific prediction results of different subtasks.

The Binary Cross Entropy loss function (BCELoss) is commonly used in binary classification tasks, where the goal is to predict a binary outcome (e.g., positive or negative). In the case of multitask learning, where there are multiple subtasks to be predicted, the BCELoss function can be used to calculate the loss for each subtask separately and then averaged to obtain the overall loss for the multitask model. The detailed Loss is expressed as follows:

$$\left.Loss=-\frac{1}{n}\sum_{i=1}^{n}\left({Label}_{i}\right.\times {logPred}_{i}+\left(1-{Label}_{i}\right)\times \log\;\left(1-{Pred}_{i}\right)\right)$$ (1)

Where, *n* is the number of training molecules in each batch; *Label* is the real Label of the molecule. *Pred* is the molecular prediction result. For multi-task prediction, the loss function calculates the loss of each subtask and takes the average value as the total loss function of multitask.

### 2.3 The baseline machine learning and deep learning algorithms

We constructed fingerprint- and graph-based models (Supplementary Table S1) to fairly compare the multi-task FP-GNN model in the CYPs inhibitors prediction tasks. Fingerprint-based prediction models were constructed based on the Morgan fingerprint (similar to ECFP, 1,024 bits) using four CML algorithms, i.e., Naive Bayes (NB) (Duda and Hart, 1973), random forest (RF) (Svetnik et al., 2003), support vector machine (SVM) (Zernov et al., 2003), and extreme gradient boosting (XGBoost) (Chen and Guestrin, 2016) and one DL method, deep neural networks (DNN) (McCulloch and Pitts, 1943). Two DL algorithms were used to create graph-based prediction models, i.e., graph attention network (GAT) (Veličković et al., 2018) and graph convolutional networks (GCN) (Kipf and Welling, 2017). A basic overview of these CML and DL techniques can be obtained elsewhere (Wu et al., 2017; He et al., 2021). All these CML and DL models, as well as FP-GNN models presented here were trained on the CPU (Intel(R) Xeon(R) Silver 4216 CPU @ 2.10 GHz) and GPU (NVIDIA Corporation GV100GL [Tesla V100 PCIe 32 GB]). Meanwhile, we compared the multi-task FP-GNN model to reported models, such as SuperCYPsPred (Banerjee et al., 2020) and iCYP-MFE (Nguyen-Vo et al., 2021).

### 2.4 Performance evaluation of models

The performance of the multi-task FP-GNN model, the baseline CML and DL models were evaluated using the following four metrics: the area under the receiver operating characteristic (AUC), F1-measure (F1 score), Matthews correlation coefficient (MCC), and balanced accuracy (BA). To evaluate the effectiveness of classification models (Niijima et al., 2012; Li et al., 2020; Jiang et al., 2021; Nguyen-Vo et al., 2021), we also used the AUC value to optimize and choose the best models. Such metrics are defined as follows:

$$F1=\frac{2\times Precision\times Recall}{Precision+Recall}=\frac{2\times TP}{2\times TP+FN+FP}$$ (2)

$$MCC=\frac{TP\times TN-FN\times FP}{\sqrt{\left(TP+FN\right)\times \left(TP+FP\right)\times \left(TN+FN\right)\times \left(TN+FP\right)}}$$ (3)

$$BA=\frac{TPR+TNR}{2}=\frac{SE+SP}{2}$$ (4)

$$SP=TNR=\frac{TN}{TN+FP}$$ (5)

$$SE=TPR=Recall=\frac{TP}{TP+FN}$$ (6)

where TP, TN, FP, FN, SP, SE, TNR, and TPR represent the number of true positives, true negatives, false positives, false negatives, specificity, sensitivity, true negative rate, and true positive rate respectively.

### 2.5 Model applicability domain

The applicability domain (AD) analysis helps us to figure out whether the built QSAR model can be applied to any set of compounds (Peter et al., 2019). For AD analysis, we used the Euclidean distance-based method (DM), which is based on structural similarity. Here is the detailed formula:

$$D_{T}=d_{ave}+Z\times \theta$$ (7)

where *d*_*ave*_ is the average Euclidean distance between each compound in the training set and its nearest k compounds. *θ* is the corresponding standard deviation. *Z* is an optional parameter representing the significance level. First, RDKit software is used to calculate the Pharmacophore ErG, PubChem, and MACCS fingerprints of the test and training sets, and then the average of the Euclidean distance is calculated. For each molecule in the training set, *d*_*ave*_ and *θ* are calculated from the Euclidean distances of the k nearest neighbors. Finally, the Euclidean distance between each molecule in the test set and the nearest neighbor molecule in the training set is determined. The compound is regarded to be outside the domain (OD) if the distance exceeds the threshold of *D*_*T*_. Otherwise, it has entered the inside domain (ID). We utilize the test set to discover acceptable parameters k and Z, and then compute the threshold of the AD of the model.

## 3 Results and discussion

### 3.1 Datasets analysis and model construction

**FIGURE 2:**
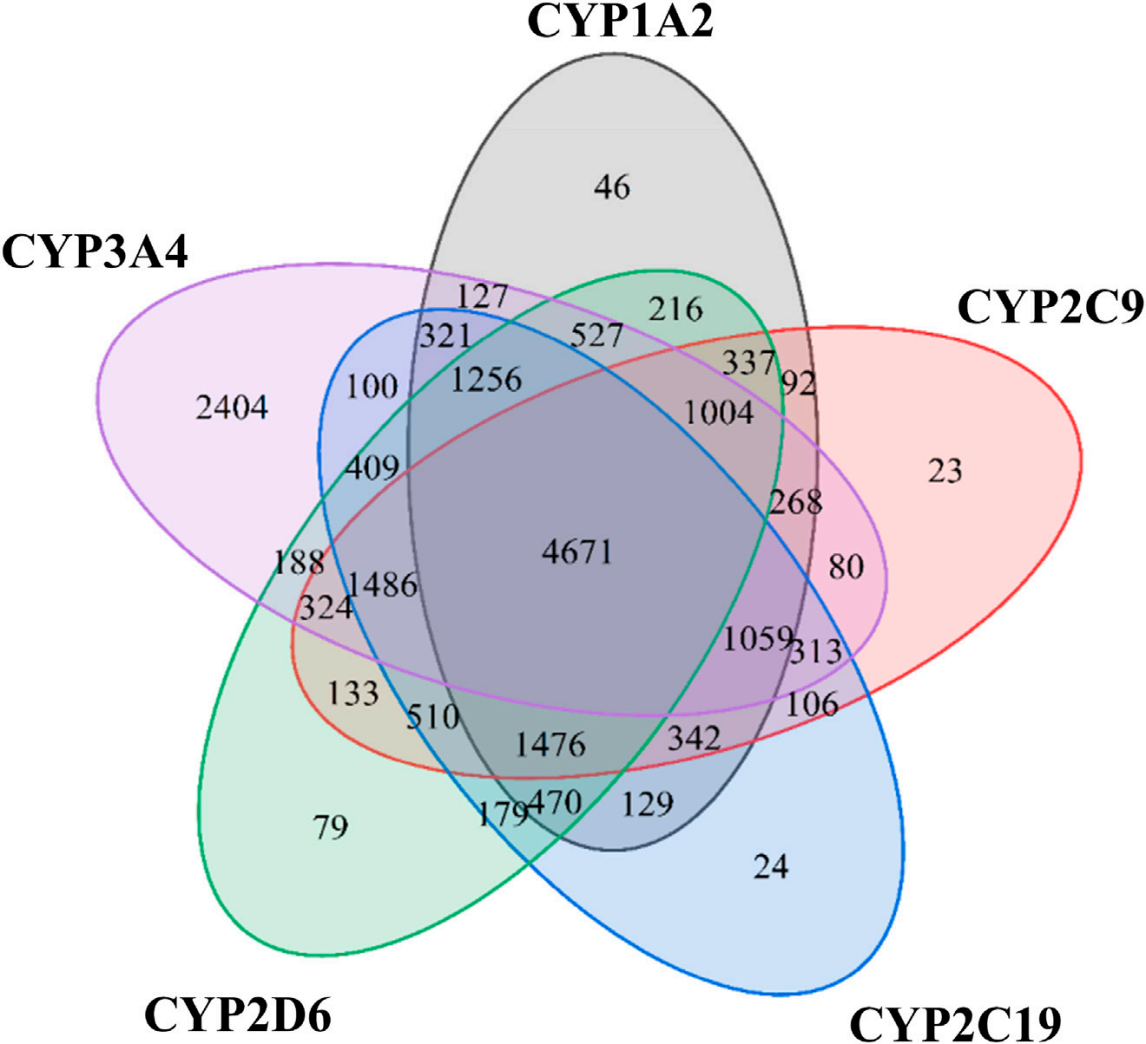
The data occupation distribution for the five isoforms in the CYPs modelling datasets.

**FIGURE 3:**
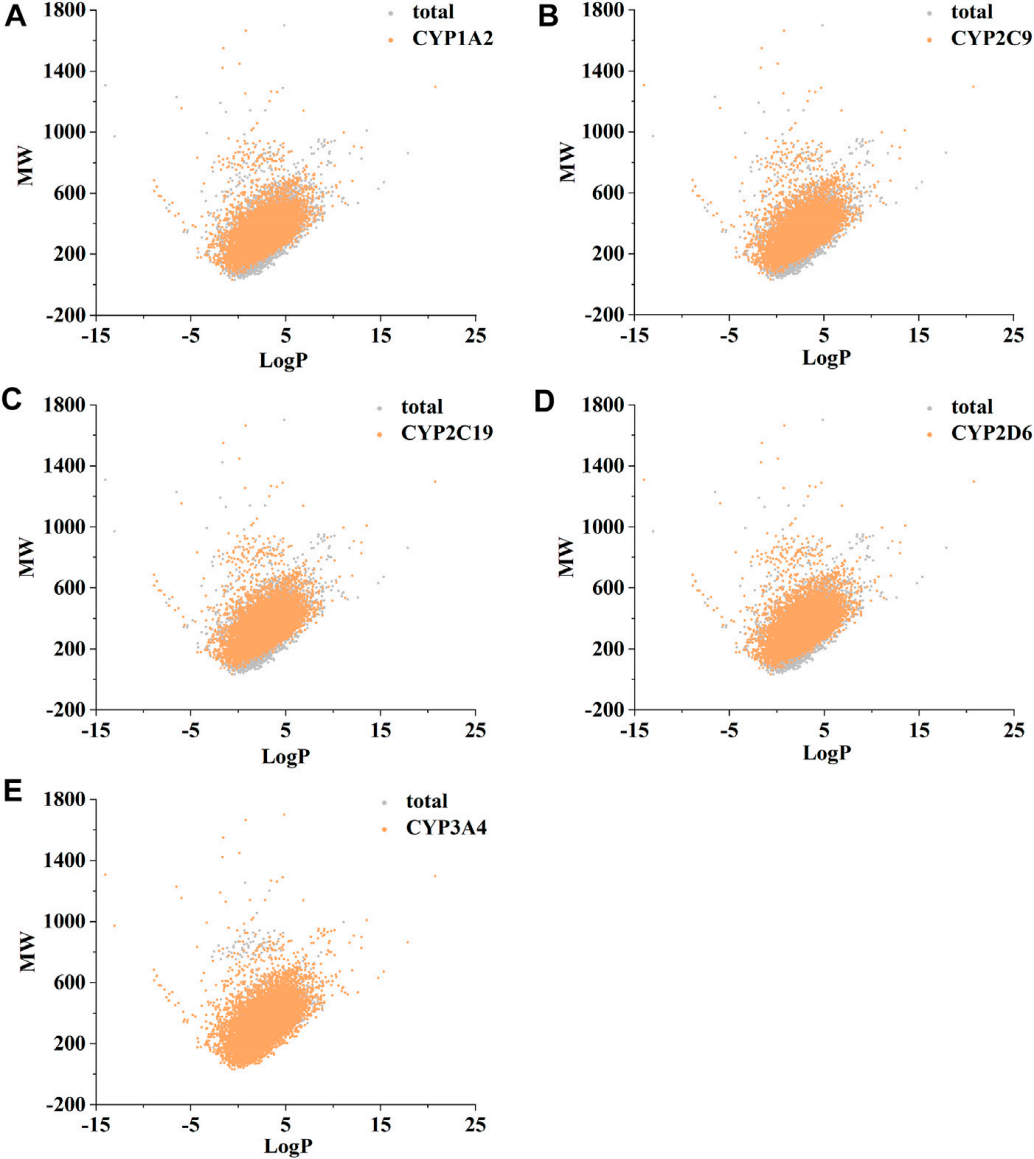
The distribution of molecular chemical space of CYP1A2 **(A)**, CYP2C9 **(B)**, CYP2C19 **(C)**, CYP2D6 **(D)**, and CYP3A4 **(E)**. LogP (*X-axis*) and molecular weight (MW, *Y-axis*) were used to define chemical space. RDKit software was used to calculate MW and LogP.

**FIGURE 4:**
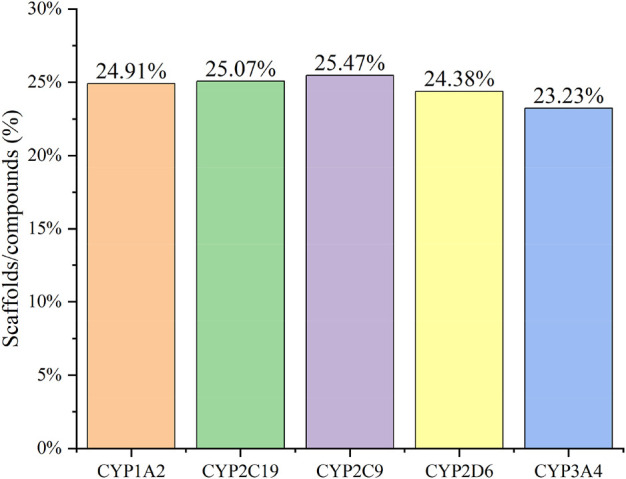
The Bemis Murcko scaffold analysis of CYP1A2 (orange), CYP2C19 (green), CYP2C9 (purple), CYP2D6 (yellow), and CYP3A4 (blue) inhibitors. The five CYPs isoforms (*X-axis*) and the fraction of scaffolds in the modeling datasets (scaffolds/compounds (%), *Y-axis*) were used to determine structural diversity.

Figure 2 confirms strong correlations between the datasets of the five isoforms used for modelling, as they share a large number of common molecular entities. As shown in Figure 3, the compounds in the CYPs modelling datasets were dispersed over a wide range of molecular weight (32.042–1701.206) and LogP (−15.231–20.751), indicating that the compounds in the modelling datasets have a vast chemical space. Meanwhile, each isoform had a comparable distribution compared to the total dataset, indicating that the five CYP isoforms are closely linked and suitable for multitasking modelling processing. Furthermore, as shown in Figure 4, Bemis Murcko scaffold (Bemis and Murcko, 1996) analysis revealed that the fraction of scaffolds in the modeling datasets ranged from 22.33% to 25.47%, showing a significant structural diversity of compounds among the five CYP subtypes.

### 3.2 Performance of the multi-task FP-GNN model on CYPs datasets

The comparison results of the multi-task FP-GNN model with other advanced CML, DL, and reported models are shown in Table 1; Supplementary Tables S2–S4. Table 1; Supplementary Tables S2–S4 illustrate that the multi-task FP-GNN model achieves the best overall performance on these five CYP isoforms, with the highest average AUC (0.905), F1 (0.779), BA (0.819), and MCC (0.647) values for the test sets. Specifically, taking the AUC value as the main evaluation metric, in four of the five subtypes (CYP1A2, CYP2C9, CYP2C19, and CYP3A4), the multi-task FP-GNN model ranked first in terms of predictive performance. Meanwhile, the multi-task FP-GNN model achieved second-ranked predictive performance on CYP2D6 (AUC = 0.883). The single-task and multitask models of iCYP-MFE have the best performance on CYP2D6, while SuperCYPsPred based on the Morgan fingerprints performed as well on CYP2C19 as the multi-task FP-GNN model. In addition, Supplementary Tables S2–S4 indicate that the multi-task FP-GNN model achieves the best-performance on other metrics. For example, in three of the five subtypes (CYP2C9, CYP2C19, and CYP3A4), the multi-task FP-GNN model ranked first in terms of F1, BA, and MCC values. Such results show that, compared with the current advanced CML, DL, as well as the existing multi-task models, the multi-task FP-GNN model presented here exhibits the state-of-the-art (SOTA) performance in predicting the inhibitory activity of compounds against the five CYPs isoforms. Furthermore, the compounds from the test set were not mispredicted for all targets (Supplementary Figure S1). The optimal set of hyperparameters for each CYP isoform is provided in Supplementary Table S5.

**TABLE 1:** The AUC value of FP-GNN on CYPs dataset compared to other baseline models.

Model	CYP1A2	CYP2C9	CYP2C19	CYP2D6	CYP3A4	AVE
SuperCYP-MACCS^31^	0.820	0.790	0.880	0.880	0.870	0.848
SuperCYP-Morgan^31^	0.830	0.870	**0.900**	0.880	0.880	0.872
iCYP-MFE (single)^19^	0.900	0.850	0.860	**0.930**	0.880	0.884
iCYP-MFE (multi)^19^	0.910	0.890	0.860	**0.930**	0.890	0.896
DNN::Morgan	0.904	0.878	0.887	0.848	0.883	0.880
RF::Morgan	0.910	0.891	0.881	0.867	0.891	0.888
SVM::Morgan	0.909	0.856	0.898	0.838	0.884	0.877
NB::Morgan	0.848	0.826	0.822	0.816	0.829	0.828
GCN	0.921	0.860	0.886	0.875	0.900	0.889
XGB::Morgan	0.888	0.857	0.868	0.842	0.864	0.864
GAT	0.928	0.888	0.885	0.861	0.896	0.891
FP-GNN (single)	0.928	0.893	0.879	0.881	0.907	0.897
FP-GNN (multi)	**0.930**	**0.902**	**0.900**	0.883	**0.911**	**0.905**

Bold font illustrates the models that outperformed all other models.

In addition to the multi-task FP-GNN model, the single-task FP-GNN model also exhibits good and/or comparable performance results, achieving the second-ranked overall predictive performance on the CYPs modelling datasets with higher average AUC (0.897), F1 (0.773), BA (0.812), and MCC (0.631) values. Specifically, the single-task FP-GNN model performed best on three CYP isoforms (CYP1A2, CYP2C9, and CYP3A4) compared to other CML, DL, as well as the existing multi-task models. Clearly, the FP-GNN model without the multi-task module still showed superior prediction performance on these five CYPs isoforms, indicating the superiority of the FP-GNN DL algorithm.

Although the FP-GNN model showed remarkable predictive performance in both single-task and multi-task models, the multi-task FP-GNN model outperformed the single-task FP-GNN model in CYPs inhibitors prediction task. The five CYPs isoforms datasets are highly correlated (Figure 1), and the multi-task FP-GNN model can capture relevant information among subtasks, thereby significantly improving the performance of the model.

Y-scrambling testing was used to demonstrate that the results were not attributed to chance correlation. As illustrated in Supplementary Figure S2, the AUC values of the multi-task FP-GNN model were significantly higher than those of any of the Y-scrambled models, confirming that the results were not chance correlations.

### 3.3 Model applicability domain

The amounts of compounds outside the AD in the test sets at different Z and k values are shown in Supplementary Table S6. It can be shown that when Z values increased and k remained constant, the number of compounds outside the AD decreased. Afterward, the multi-task FP-GNN model was used to predict the ID and OD chemicals in the test sets at various k and Z values, and the detailed performance of each data set is presented in Supplementary Table S7. We found that when k = 3, *Z* = 0.2, the overall evaluation metrics of the model were improved, and it was able to discriminate between ID and OD compounds of the CYP datasets to the maximum extent. The predictive performance of ID compounds (AUC = 0.920, F1 = 0.804, BA = 0.842, and MCC = 0.689) was significantly better than that of OD compounds (AUC = 0.852, F1 = 0.692, BA = 0.748, and MCC = 0.510). The results showed that our defined AD is appropriate for the suggested multi-task FP-GNN model and it might help the model serve more properly in real-world situations.

### 3.4 Interpretation of the multi-task FP-GNN model

**FIGURE 5:**
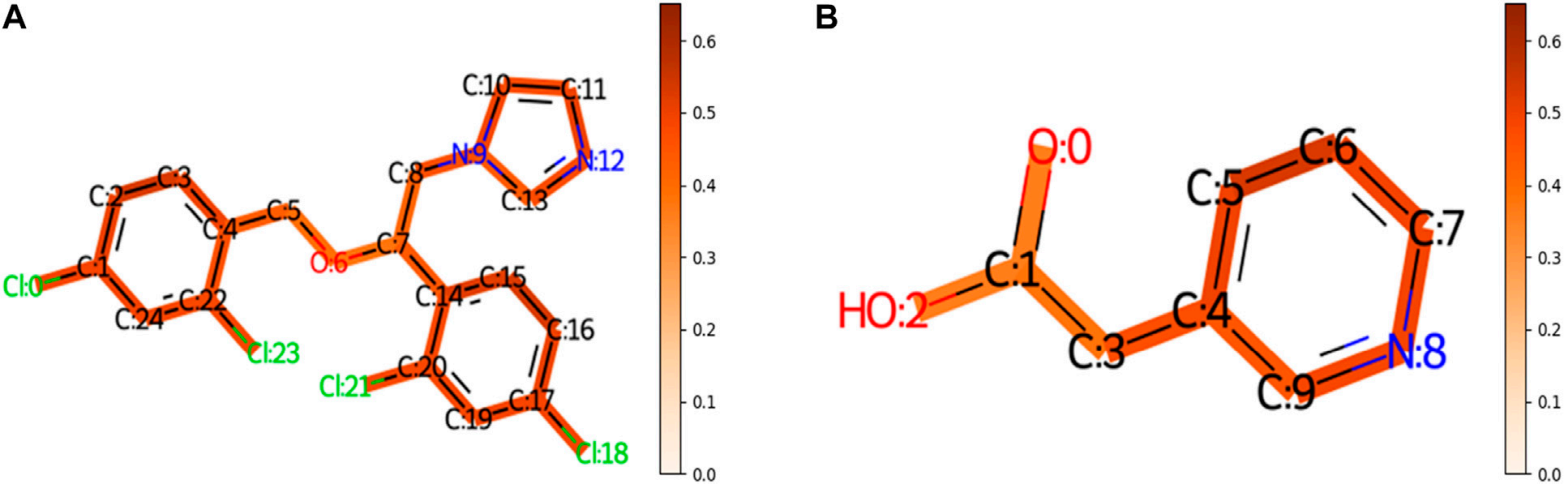
The importance of molecular structures during the prediction process of the GNN module of the multi-task FP-GNN model on the CYPs dataset. The darker the color, the more important are for the structures. **(A)** Represents an active molecule on the five isoforms. **(B)** Represents an inactive molecule on the five isoforms.

To understand the multi-task FP-GNN model for the prediction of CYP inhibitors, we accomplished an interpretation of its GNN and FPN modules. Taking an active molecule (Miconazole, CHEMBL91, Figure 5A) and an inactive molecule (Figure 5B) as examples, the multi-task FP-GNN architecture can calculate the attention coefficients of neighboring atoms and map them to the bonds that connect them. Chemical fragments contribute more to the prediction of CYPs inhibitory activity when the attention coefficient for the molecule is higher. In other words, the portions of the molecule colored more darkly were more essential in predicting whether the molecule can inhibit CYPs, and *vice versa*.

In addition to the GNN module, we also investigated the interpretation of the FPN module on the CYP modelling datasets. Table 2 summarizes the top ten most significant bits, which represent important structural fragments or pharmacophore feature information that contribute greatly to the inhibitory activity of CYPs. Collectively, these fragments may facilitate in the design and optimization of novel CYPs inhibitors.

**TABLE 2:** The top ten significant bits from the FPN module of the multi-task FP-GNN model on the CYPs datasets.

Rank	Importance	Mixed FP Bit	FP Class	Meaning
1	0.00210	35	MACCS	CH2 = A
2	0.00184	31	MACCS	CQ(C) (C)A
3	0.00180	489	Pharmacophore ErG	(‘Negative’, ‘Negative’, 7)
4	0.00177	493	Pharmacophore ErG	(‘Negative’, ‘Negative’, 11)
5	0.00173	640	PubChem	>= 2 P
6	0.00164	369	Pharmacophore ErG	(‘Acceptor’, ‘Hydrophobic’, 13)
7	0.00153	956	PubChem	C (∼C) (∼H) (∼O) (∼O)
8	0.00139	371	Pharmacophore ErG	(‘Acceptor’, ‘Hydrophobic’, 15)
9	0.00122	612	PubChem	>= 32 H
10	0.00119	32	MACCS	QX

Q: atom of non-C, or non-H.

X: atom of other than H, C, N, O, Si, P, S, F, Cl, Br, I.

A: any valid periodic table element symbol.

### 3.5 Webserver construction and use

**FIGURE 6:**
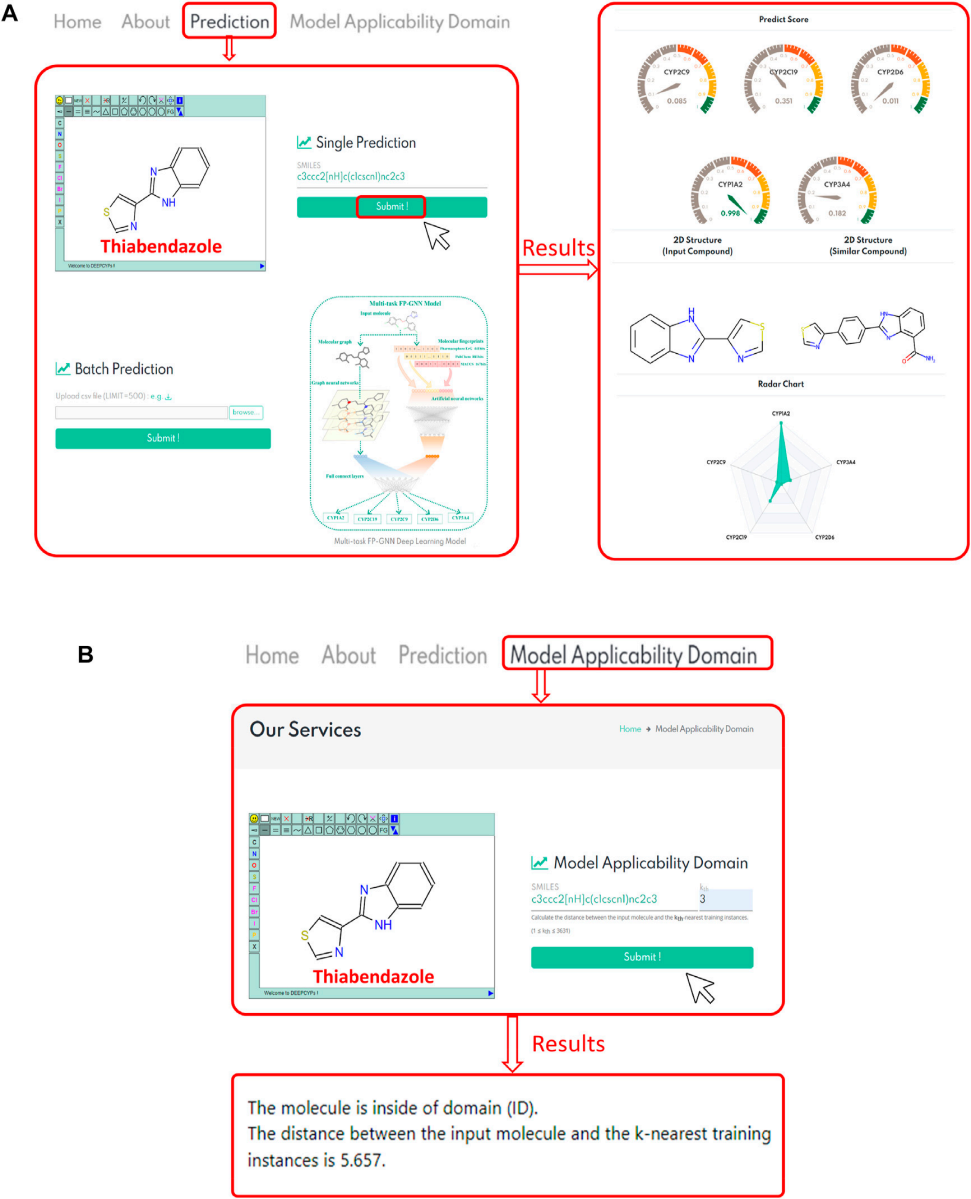
**(A)** Represents the bioactivity prediction diagram of the DEEPCYPs. **(B)** Represents a case result display of the model applicability domain module of the DEEPCYPs. The chemical structure of thiabendazole is used as an example.

DEEPCYPs, an online platform for the prediction of cytochrome activity, was constructed based on the established multitask FP-GNN model. Users can draw a structure online, input or upload structures in SMILES format to conveniently predict the inhibitory activity and selectivity of molecules against five major CYP isoforms (e.g., CYP1A2, CYP2C19, CYP2C9, CYP2D6, and CYP3A4) (Figure 6A, left). Existing machine learning-based predictive models, including our multi-task FP-GNN model, are classification models that can only assess the likelihood of inhibiting CYPs (i.e., probability score, 0–1) for compounds of interest. The 0.5 threshold is used to determine whether or not a molecule inhibits CYP. Based on the predicted score, DEEPCYPs can be used to assess the relative inhibitory potential of compounds against specific CYP subtypes. The higher the score, the more likely it is that the subtype will be suppressed.

(Banerjee et al., 2020) constructed SuperCYP using the RF model with two types of molecular fingerprints (Morgan and MACCS). iCYP-MFE (Nguyen-Vo et al., 2021) was developed by multi-task convolutional neural networks combined with molecular fingerprint embedding features. Compared to SuperCYP, developing the DEEPCYPs dataset is larger than SuperCYP, which is important for the improvement of the model performance. Compared with SuperCYP and iCYP-MFE, DEEPCYP can not only learn effective and complementary information from molecular graph and molecular fingerprints but also learn the information dependent on each other in relevant data sets, which is beneficial to improve the prediction accuracy of the model. Furthermore, unlike SuperCYP and iCYP-MFE, users can obtain the distance between the input molecule and the k-nearest training instances on the Model Applicability Domain module of the DEEPCYPs (Figure 6B). Detailed comparison of DEEPCYPs with the advanced existing models such as SuperCYP and iCYP-MFE is shown in Table 3. Clearly, DEEPCYPs shows advantages in terms of accuracy, functionality and ease of use.

**TABLE 3:** The detailed comparison of DEEPCYPs and the most advanced existing models (SuperCYP and iCYP-MFE).

Model	Average values			Interpretation	Model applicability domain	Webserver
	AUC^a^	F1^b^	BA^c^			
DEEPCYPs (Our)	**0.905**	**0.779**	**0.819**	**Yes**	**Yes**	■ Input format: draw a structure online, input or upload structures in SMILES.
						■ Prediction: realize both single-molecule prediction and batch-molecules prediction simultaneously
						■ Model Applicability Domain: users can get the distance between the input molecule and the k-nearest training instances
iCYP-MFE (multi)	0.896	0.754	0.796	No	No	Yes, but not work
SuperCYP-MACCS	0.848	0.572	0.710	No	No	■ Input format: input structures in SMILES.
SuperCYP-Morgan	0.872	0.538	0.704			■ Prediction: single-molecule prediction

^a^AUC: The area under receiver operating characteristic.

^b^F1: F1-measure.

^c^BA: balanced accuracy.

To validate website prediction performance, we chose molecules within the dataset (thiabendazole and fenofibrate) and molecules outside the dataset (quinidine and telithromycin) that have been reported to be CYP-related inhibitors. Taking thiabendazole as an example (Figure 6A, right), it has a predicted score of 0.998 in the CYP1A2 model, indicating that it has a strong inhibitory effect on the CYP1A2 isoform. Indeed, thiabendazole is an effective and specific inhibitor of CYP1A2 (IC_50_ = 0.830 μM) (Thelingwani et al., 2009), proving the accuracy and usability of the DEEPCYPs webserver. In addition, the bioactivity prediction results of quinidine (a potent CYP2D6 inhibitor, IC_50_ = 0.156 μM) (McLaughlin et al., 2005; Kang et al., 2019), telithromycin (a potent CYP3A4 inhibitor, IC_50_ = 11.800 μM) (Li et al., 2019), and fenofibrate (an effective CYP2C19/2C9 inhibitor; CYP2C19, IC_50_ = 0.200 μM; CYP2C9, IC_50_ = 9.700 μM) (Schelleman et al., 2014) by the DEEPCYPs are shown in Supplementary Figure S3. The predicted results are generally consistent with real-world drug inhibitory effects, indicating that the DEEPCYPs webserver can not only predict whether the compound has an inhibitory effect on individual CYP450 isoform but also predict whether the compound is selective for dual CYP450 isoforms. However, we must declare that DEEPCYPs can only give prediction results, but it does not mean that the predictions are correct. The predictions can be combined with experiments for further verification. The presence of predictive models is that large-scale compound libraries can be quickly evaluated, and models can outline which chemical fragments are more likely to produce CYP inhibitors, which can help optimize subsequent lead compounds.

## 4 Conclusion

The multi-task FP-GNN was used for the prediction of CYPs inhibitors, which outperformed the baseline models, such as Morgan fingerprint-based ML models (i.e., NB, RF, SVM, XGBoost, and DNN), graph-based DL models (i.e., GAT and GCN), and current reported models (i.e., SuperCYP and iCYP-MFE). Therefore, we constructed DEEPCYPs, a user-friendly webserver for predicting the inhibitory activity of molecules against the five CYP isoforms (CYP1A2, CYP2C9, CYP2C19, CYP2D6, and CYP3A4) based on the multi-task FP-GNN model. We anticipate that DEEPCYPs and its python software can support scientific communities in prioritizing molecules in drug discovery practice and/or identifying CYP inhibitors.

## Data Availability

The original contributions presented in the study are included in the article/Supplementary Material, further inquiries can be directed to the corresponding author.
