# Supplementary material for: DEEPCYPs: A deep learning platform for enhanced cytochrome P450 activity prediction
Source: Front Pharmacol. 2023 Apr 10;14:1099093. doi: 10.3389/fphar.2023.1099093 (PMC10123292; doi:10.3389/fphar.2023.1099093)
Supplement: Supplementary file 1 [file Table1.DOCX]

Supplementary Material

**DEEPCYPs: an online platform for cytochrome P450**

**activity prediction**

Daiqiao Ai^a, ‡^, Hanxuan Cai^a, ‡^, Jiajia Wei^a, ‡^, Duancheng Zhao^a^, Yihao Chen^a^, Ling Wang^a,^ *

*^a^Guangdong Provincial Key Laboratory of Fermentation and Enzyme Engineering, Joint International Research Laboratory of Synthetic Biology and Medicine, Guangdong Provincial Engineering and Technology Research Center of Biopharmaceuticals, School of Biology and Biological Engineering, South China University of Technology, Guangzhou 510006, China.*

*^‡^These authors contributed equally to this work*

**E-mail:* [*lingwang@scut.edu.cn*](mailto:lingwang@scut.edu.cn)

**Contents**

**Supplementary Figure S1.** The data occupation for the mispredicted compounds for all targets from the test set.

**Supplementary Figure S2.** The Y-scrambling results for the multi-task FP-GNN model on the CYPs datasets. Original_data were evaluated on the original test sets, while others were evaluated on the test sets changed randomly with different seeds.

**Supplementary Figure S3.** The bioactivity prediction results of quinidine, telithromycin, and fenofibrate by the DEEPCYPs.

**Supplementary Table S1.** The details of machine learning algorithms and model construction.

**Supplementary Table S2.** The F1 value of FP-GNN on CYPs dataset compared to other baseline models.

**Supplementary Table S3.** The BA value of FP-GNN on CYPs dataset compared to other baseline models.

**Supplementary Table S4.** The MCC value of FP-GNN on CYPs dataset compared to other baseline models.

**Supplementary Table S5.** The optimal set of hyperparameters for each of the CYP isoforms.

**Supplementary Table S6.** The amounts of compounds outside the applicability domain in the test set at different Z and k values.

**Supplementary Table S7.** The evaluation performance of compounds both inside domain (ID) and outside the domain (OD) in the test set at different Z and *k* values.


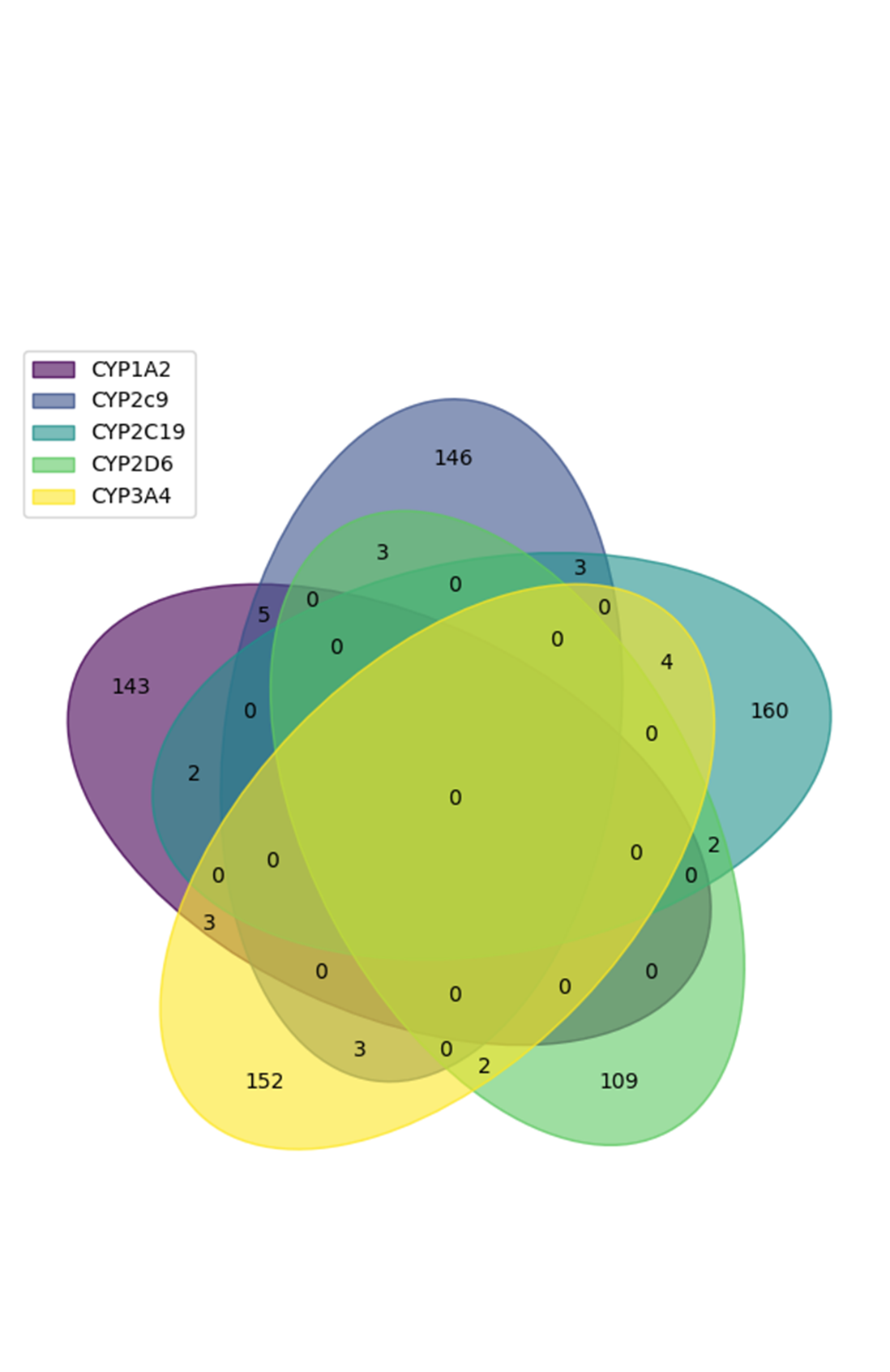


**Supplementary Figure S1.** The data occupation for the mispredicted compounds for all targets from the test set.





**Supplementary Figure S2.** The Y-scrambling results for the multi-task FP-GNN model on the CYPs datasets. Original_data were evaluated on the original test sets, while others were evaluated on the test sets changed randomly with different seeds.

**
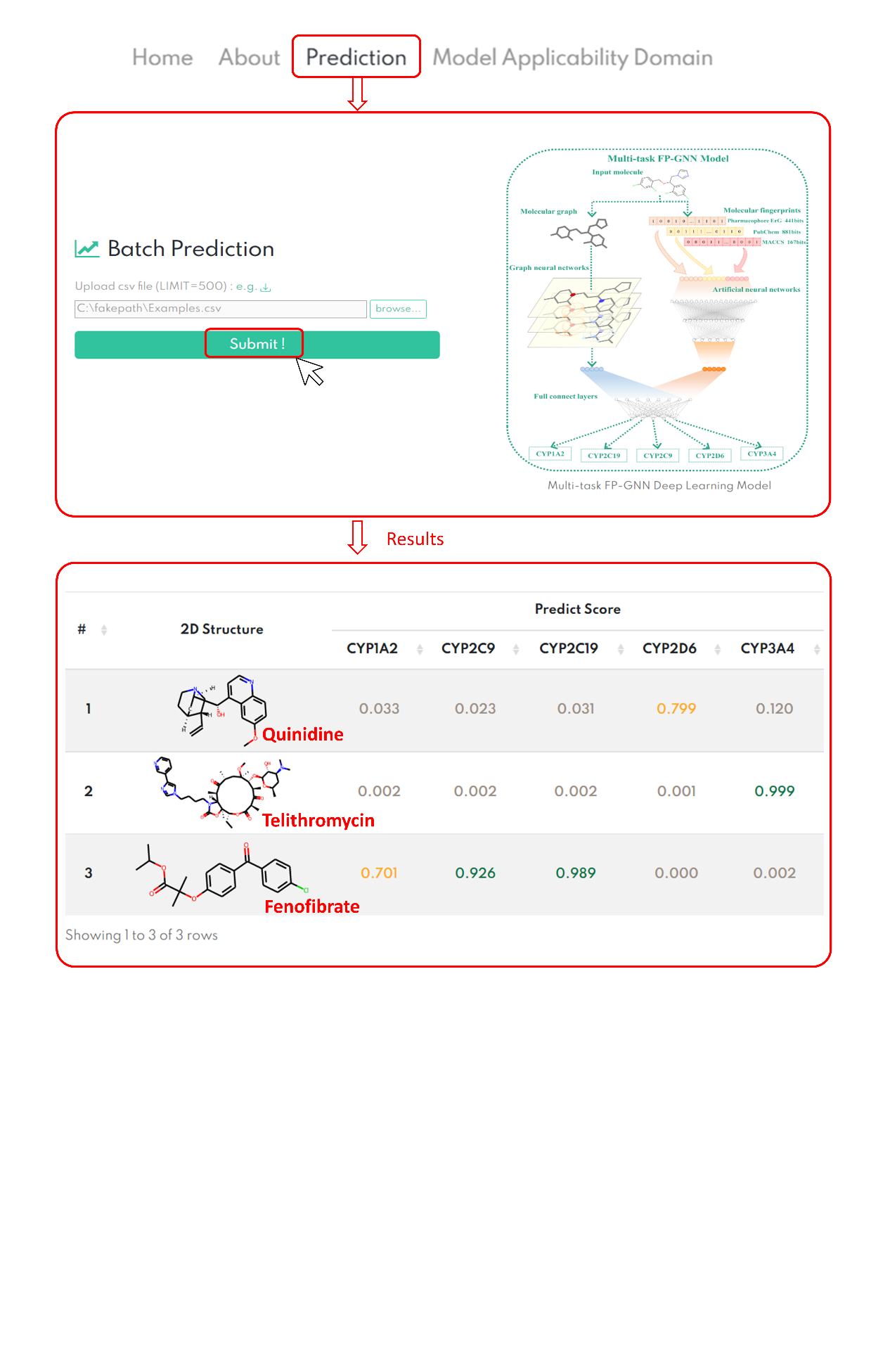
**

**Supplementary Figure S3.** The bioactivity prediction results of quinidine, telithromycin, and fenofibrate by the DEEPCYPs.

**Supplementary Table S1.** The details of machine learning algorithms and model construction.

| **Feature** | **Model** | **Package** | **Link** |
| --- | --- | --- | --- |
| Morgan fingerprint | RF | scikit-learn | https://github.com/scikit-learn/scikit-learn |
| Morgan fingerprint | SVM | scikit-learn | https://github.com/scikit-learn/scikit-learn |
| Morgan fingerprint | XGBoost | XGBoost | https://github.com/dmlc/xgboost |
| Morgan fingerprint | DNN | DeepChem | https://github.com/deepchem/deepchem |
| Molecular graph | GCN | DeepChem | https://github.com/deepchem/deepchem |
| Molecular graph | GAT | DeepChem | https://github.com/deepchem/deepchem |
| Fingerprints^a^ and molecular graph | FP-GNN | FP-GNN (*in-house*) | https://github.com/idrugLab/FP-GNN |

^a^Fingerprints: PubChem fingerprint, MACCS fingerprint, and Pharmacophore ErG fingerprint.

**Supplementary Table S2.** The F1 value of FP-GNN on CYPs dataset compared to other baseline models.

| **Model** | **CYP1A2** | **CYP2C9** | **CYP2C19** | **CYP2D6** | **CYP3A4** | **AVE** |
| --- | --- | --- | --- | --- | --- | --- |
| SuperCYP-MACCS | 0.530 | 0.570 | 0.490 | 0.610 | 0.660 | 0.572 |
| SuperCYP-Morgan | 0.560 | 0.370 | 0.430 | 0.610 | 0.720 | 0.538 |
| iCYP-MFE (single) | 0.810 | 0.680 | 0.750 | 0.690 | 0.720 | 0.730 |
| iCYP-MFE (multi) | 0.830 | 0.700 | 0.760 | 0.700 | 0.780 | 0.754 |
| DNN::Morgan | 0.815 | 0.700 | 0.800 | 0.549 | 0.752 | 0.723 |
| RF::Morgan | 0.821 | 0.614 | 0.787 | 0.487 | 0.687 | 0.679 |
| SVM::Morgan | 0.821 | 0.660 | 0.809 | 0.499 | 0.762 | 0.710 |
| NB::Morgan | 0.748 | 0.664 | 0.734 | 0.519 | 0.694 | 0.672 |
| GCN | 0.824 | 0.710 | 0.780 | 0.599 | 0.750 | 0.732 |
| XGB::Morgan | 0.787 | 0.623 | 0.783 | 0.505 | 0.725 | 0.685 |
| GAT | 0.846 | 0.744 | 0.786 | 0.539 | 0.773 | 0.738 |
| FP-GNN (single) | **0.857** | 0.728 | 0.789 | **0.693** | 0.800 | 0.773 |
| FP-GNN (multi) | 0.855 | **0.758** | **0.820** | 0.646 | **0.814** | **0.779** |

**Supplementary Table S3**. The BA value of FP-GNN on CYPs dataset compared to other baseline models.

| **Model** | **CYP1A2** | **CYP2C9** | **CYP2C19** | **CYP2D6** | **CYP3A4** | **AVE** |
| --- | --- | --- | --- | --- | --- | --- |
| SuperCYP-MACCS | 0.670 | 0.680 | 0.650 | **0.810** | 0.740 | 0.710 |
| SuperCYP-Morgan | 0.690 | 0.610 | 0.640 | **0.810** | 0.770 | 0.704 |
| iCYP-MFE (single) | 0.810 | 0.760 | 0.780 | 0.780 | 0.770 | 0.780 |
| iCYP-MFE (multi) | 0.820 | 0.780 | 0.780 | 0.800 | 0.800 | 0.796 |
| DNN::Morgan | 0.825 | 0.774 | 0.813 | 0.717 | 0.795 | 0.785 |
| RF::Morgan | 0.831 | 0.719 | 0.805 | 0.664 | 0.753 | 0.754 |
| SVM::Morgan | 0.832 | 0.747 | 0.825 | 0.673 | 0.802 | 0.776 |
| NB::Morgan | 0.761 | 0.751 | 0.748 | 0.715 | 0.743 | 0.744 |
| GCN | 0.834 | 0.785 | 0.802 | 0.734 | 0.795 | 0.790 |
| XGB::Morgan | 0.799 | 0.723 | 0.800 | 0.678 | 0.774 | 0.755 |
| GAT | 0.840 | 0.812 | 0.806 | 0.694 | 0.810 | 0.793 |
| FP-GNN (single) | **0.853** | 0.795 | 0.794 | 0.797 | 0.821 | 0.812 |
| FP-GNN (multi) | 0.846 | **0.815** | **0.828** | 0.772 | **0.834** | **0.819** |

**Supplementary Table S4**. The MCC value of FP-GNN on CYPs dataset compared to other baseline models.

| **Model** | **CYP1A2** | **CYP2C9** | **CYP2C19** | **CYP2D6** | **CYP3A4** | **AVE** |
| --- | --- | --- | --- | --- | --- | --- |
| SuperCYP-MACCS | 0.420 | 0.350 | 0.390 | 0.530 | 0.520 | 0.442 |
| SuperCYP-Morgan | 0.460 | 0.380 | 0.400 | 0.530 | 0.560 | 0.466 |
| iCYP-MFE (single) | 0.620 | 0.550 | 0.560 | **0.660** | 0.570 | 0.592 |
| iCYP-MFE (multi) | 0.640 | 0.580 | 0.570 | 0.640 | 0.600 | 0.606 |
| DNN::Morgan | 0.651 | 0.573 | 0.623 | 0.460 | 0.601 | 0.582 |
| RF::Morgan | 0.663 | 0.523 | 0.610 | 0.485 | 0.547 | 0.565 |
| SVM::Morgan | 0.666 | 0.525 | 0.647 | 0.469 | 0.603 | 0.582 |
| NB::Morgan | 0.523 | 0.484 | 0.494 | 0.404 | 0.481 | 0.477 |
| GCN | 0.669 | 0.564 | 0.608 | 0.538 | 0.610 | 0.598 |
| XGB::Morgan | 0.601 | 0.497 | 0.599 | 0.459 | 0.562 | 0.544 |
| GAT | 0.688 | 0.614 | 0.612 | 0.501 | 0.616 | 0.606 |
| FP-GNN (single) | **0.704** | 0.588 | 0.588 | 0.637 | 0.639 | 0.631 |
| FP-GNN (multi) | 0.693 | **0.639** | **0.657** | 0.580 | **0.668** | **0.647** |

**Supplementary Table S5**. The optimal set of hyperparameters for each of the CYP isoforms.

| Methods | Target | Hyperparameters | | | | | |
| --- | --- | --- | --- | --- | --- | --- | --- |
|  |  | dropout | dropout_gat | fp_2_dim | gat_scale | nheads | nhid |
| FP-GNN (single) | CYP1A2 | 0.450 | 0.350 | 350 | 0.700 | 3 | 65 |
|  | CYP2C9 | 0.500 | 0.600 | 600 | 0.300 | 8 | 70 |
|  | CYP2C19 | 0.000 | 0.100 | 600 | 0.800 | 3 | 40 |
|  | CYP2D6 | 0.450 | 0.500 | 400 | 0.600 | 7 | 40 |
|  | CYP3A4 | 0.400 | 0.500 | 550 | 0.300 | 6 | 40 |
| FP-GNN (multi) | | 0.100 | 0.500 | 450 | 0.700 | 6 | 60 |

**Supplementary Table S6.** The amounts of compounds outside the applicability domain in the test set at different Z and k values.

| **Z *k*** | **-0.5** | **-0.4** | **-0.3** | **-0.2** | **0.2** | **0.3** | **0.4** | **0.5** |
| --- | --- | --- | --- | --- | --- | --- | --- | --- |
| 1 | 1649 | 1492 | 1363 | 1252 | 706 | 583 | 479 | 394 |
| 2 | 1524 | 1389 | 1261 | 1136 | 576 | 463 | 374 | 298 |
| 3 | 1460 | 1320 | 1195 | 1042 | 493 | 400 | 314 | 247 |
| 4 | 1403 | 1275 | 1141 | 978 | 437 | 348 | 278 | 207 |

**Supplementary Table S7.** The evaluation performance of compounds both inside domain (ID) and outside the domain (OD) in the test set at different Z and k values.

| **k** | **Z** | **AUC^a^** | | **F1^b^** | | **BA^c^** | | **MCC^d^** | |
| --- | --- | --- | --- | --- | --- | --- | --- | --- | --- |
|  |  | **ID** | **OD** | **ID** | **OD** | **ID** | **OD** | **ID** | **OD** |
| 1 | 0.2 | 0.918 | 0.869 | 0.802 | 0.711 | 0.840 | 0.765 | 0.687 | 0.543 |
| 1 | -0.2 | 0.920 | 0.869 | 0.805 | 0.711 | 0.843 | 0.765 | 0.691 | 0.545 |
| 1 | 0.3 | 0.919 | 0.876 | 0.802 | 0.727 | 0.842 | 0.775 | 0.688 | 0.567 |
| 1 | -0.3 | 0.919 | 0.882 | 0.801 | 0.739 | 0.840 | 0.785 | 0.686 | 0.584 |
| 1 | 0.4 | 0.914 | 0.852 | 0.791 | 0.698 | 0.830 | 0.755 | 0.667 | 0.531 |
| 1 | -0.4 | 0.910 | 0.867 | 0.786 | 0.705 | 0.826 | 0.765 | 0.658 | 0.559 |
| 1 | 0.5 | 0.910 | 0.859 | 0.786 | 0.697 | 0.826 | 0.756 | 0.658 | 0.539 |
| 1 | -0.5 | 0.908 | 0.865 | 0.783 | 0.708 | 0.824 | 0.764 | 0.655 | 0.552 |
| 2 | 0.2 | 0.919 | 0.859 | 0.803 | 0.699 | 0.841 | 0.756 | 0.688 | 0.524 |
| 2 | -0.2 | 0.918 | 0.870 | 0.802 | 0.713 | 0.840 | 0.766 | 0.686 | 0.546 |
| 2 | 0.3 | 0.920 | 0.870 | 0.804 | 0.715 | 0.843 | 0.767 | 0.690 | 0.551 |
| 2 | -0.3 | 0.920 | 0.876 | 0.803 | 0.728 | 0.842 | 0.776 | 0.689 | 0.566 |
| 2 | 0.4 | 0.910 | 0.867 | 0.786 | 0.702 | 0.826 | 0.764 | 0.658 | 0.557 |
| 2 | -0.4 | 0.909 | 0.868 | 0.785 | 0.705 | 0.825 | 0.763 | 0.657 | 0.551 |
| 2 | 0.5 | 0.908 | 0.870 | 0.784 | 0.697 | 0.824 | 0.758 | 0.655 | 0.541 |
| 2 | -0.5 | 0.907 | 0.872 | 0.782 | 0.700 | 0.822 | 0.771 | 0.652 | 0.564 |
| 3 | 0.2 | 0.920 | 0.852 | 0.804 | 0.692 | 0.842 | 0.748 | 0.689 | 0.510 |
| 3 | -0.2 | 0.919 | 0.863 | 0.803 | 0.704 | 0.841 | 0.761 | 0.687 | 0.534 |
| 3 | 0.3 | 0.919 | 0.870 | 0.803 | 0.715 | 0.841 | 0.767 | 0.687 | 0.550 |
| 3 | -0.3 | 0.920 | 0.873 | 0.803 | 0.724 | 0.843 | 0.772 | 0.690 | 0.561 |
| 3 | 0.4 | 0.910 | 0.858 | 0.786 | 0.697 | 0.826 | 0.756 | 0.659 | 0.540 |
| 3 | -0.4 | 0.908 | 0.865 | 0.784 | 0.706 | 0.824 | 0.763 | 0.655 | 0.549 |
| 3 | 0.5 | 0.907 | 0.872 | 0.782 | 0.717 | 0.822 | 0.775 | 0.652 | 0.569 |
| 3 | -0.5 | 0.907 | 0.864 | 0.782 | 0.685 | 0.822 | 0.768 | 0.651 | 0.556 |
| 4 | 0.2 | 0.918 | 0.855 | 0.798 | 0.705 | 0.838 | 0.753 | 0.682 | 0.524 |
| 4 | -0.2 | 0.919 | 0.860 | 0.803 | 0.700 | 0.840 | 0.757 | 0.687 | 0.526 |
| 4 | 0.3 | 0.919 | 0.868 | 0.802 | 0.713 | 0.840 | 0.766 | 0.686 | 0.546 |
| 4 | -0.3 | 0.920 | 0.870 | 0.804 | 0.716 | 0.843 | 0.767 | 0.691 | 0.551 |
| 4 | 0.4 | 0.909 | 0.861 | 0.785 | 0.696 | 0.825 | 0.759 | 0.656 | 0.542 |
| 4 | -0.4 | 0.908 | 0.863 | 0.784 | 0.698 | 0.824 | 0.756 | 0.655 | 0.533 |
| 4 | 0.5 | 0.907 | 0.871 | 0.782 | 0.696 | 0.822 | 0.769 | 0.652 | 0.560 |
| 4 | -0.5 | 0.906 | 0.860 | 0.781 | 0.680 | 0.820 | 0.779 | 0.649 | 0.569 |

^a^AUC: The area under receiver operating characteristic. ^b^F1: F1-measure. ^c^BA: Balanced accuracy. ^d^MCC: Matthews correlation coefficient.
